# Vortex Light Bullets Guided by Disclination Defects

**DOI:** 10.1002/nap2.70206

**Published:** 2026-07-14

**Authors:** Zhuo Zhang, Sergey K. Ivanov, Yongdong Li, Yaroslav V. Kartashov, Yiqi Zhang

**Affiliations:** ^1^ Key Laboratory for Physical Electronics and Devices Ministry of Education School of Electronic Science and Engineering Xi'an Jiaotong University Xi'an China; ^2^ Instituto de Ciencia de los Materiales Universidad de Valencia Paterna Spain; ^3^ Institute of Spectroscopy Russian Academy of Sciences Moscow Russia; ^4^ State Key Laboratory of Human‐Machine Hybrid Augmented Intelligence Institute of Artificial Intelligence and Robotics Xi'an Jiaotong University Xi'an China

**Keywords:** disclination defect, orbital angular momentum, spatial‐temporal localization, vortex light bullet

## Abstract

The formation of stable spatiotemporal optical solitons carrying vorticity, or vortex light bullets, in a nonlinear medium remains a major challenge due to the possibility of collapse in materials with ubiquitous Kerr nonlinearity and the inherent fragility of vortex‐carrying solitons to azimuthal perturbations. Although periodic optical potentials may suppress azimuthal instabilities for certain lattice configurations, vortex light bullets supported by these structures have so far been observed only as transient, weakly unstable objects. Here, we report a new class of stable, thresholdless light bullets carrying vorticity and forming in aperiodic lattices with a focusing cubic nonlinearity. These states can be guided by the disclination defects introduced into Su–Schrieffer–Heeger‐like lattices and are thresholdless since they bifurcate from linear disclination modes. Aperiodic disclination lattices considered here may possess different discrete rotational symmetry that in turn determines the allowed values of the topological charge of the vortex bullets. The resulting vortex light bullets can exist in finite spectral gaps and feature wide stability intervals. Our findings show that aperiodic lattices provide rich opportunities for the stabilization of multidimensional states and lead to nontrivial interplay between vorticity and discrete rotational symmetry of the lattice.

## Graphical abstract

A new class of stable and thresholdless light bullets carrying vorticity, guided by the disclination defects introduced into Su–Schrieffer–Heeger‐like lattices with a focusing cubic nonlinearity, is reported. Different discrete rotational symmetry of the aperiodic lattice determines the allowed values of the topological charge of the vortex bullets.

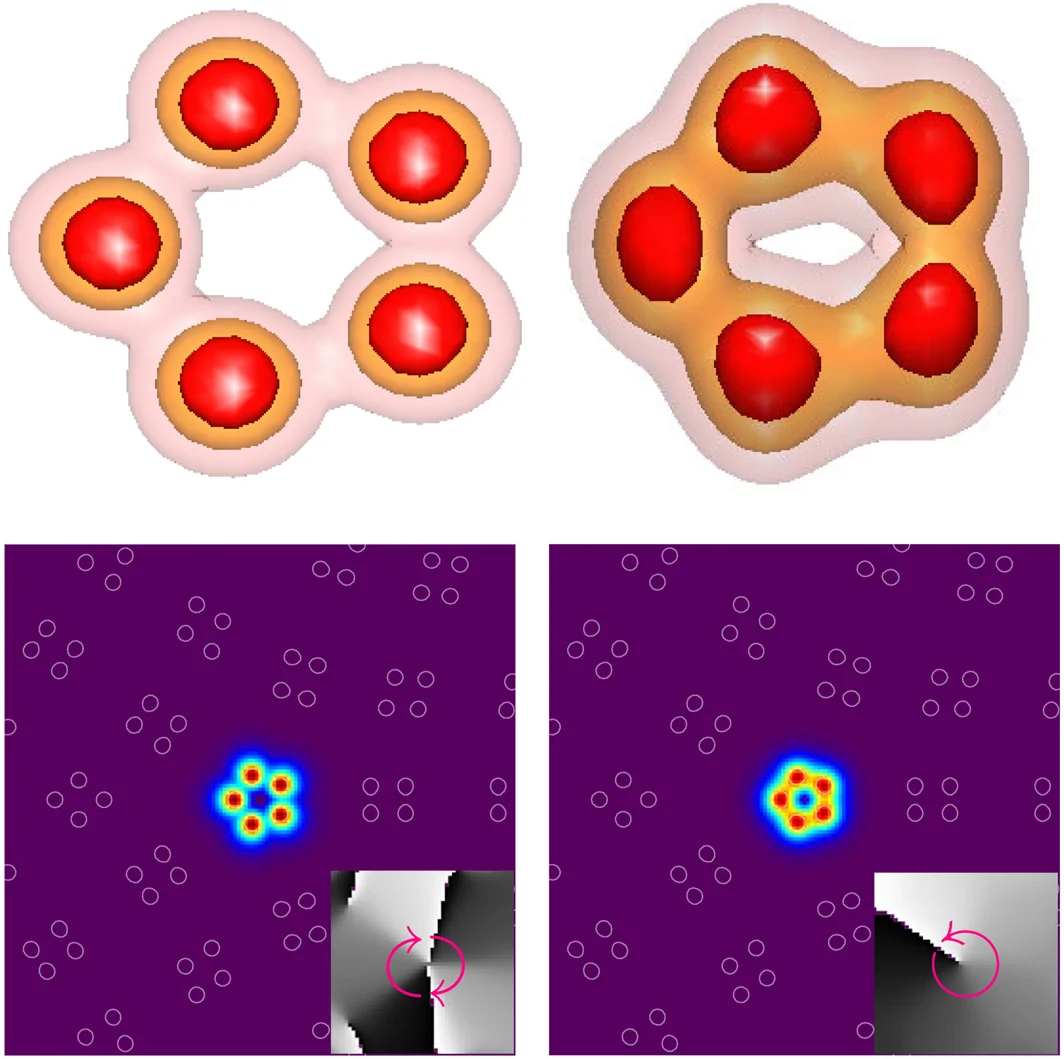

## 1 Introduction

The light bullet [1] is a three‐dimensional (3D) localized wavepacket that maintains its spatial and temporal profile during propagation in a nonlinear medium due to the simultaneous exact balance between diffraction, dispersion, and self‐action effects [2, 3, 4, 5, 6, 7]. As multidimensional nonlinear localized states capable of carrying information and energy of the light field, light bullets have attracted considerable attention in optics and have been studied in conservative materials with different types of nonlinearity, including Kerr [1, 8, 9], saturable [10], nonlocal [11, 12, 13], quadratic [14], and competing ones [15, 16, 17, 18], as well as in various dissipative systems [19] including resonator systems [20, 21, 22, 23] and lasers [24, 25, 26, 27, 28]. One of the major difficulties in experimental realization of light bullets is their instability in uniform materials with ubiquitous Kerr nonlinearity, where 3D wavepackets can undergo super‐critical collapse [29, 30]. In such materials, localized and periodic refractive index modulations, even when shallow, can potentially suppress collapse, leading to the formation of stable fundamental (vortex‐free) light bullets. This idea, originally proposed for discrete waveguide arrays [31, 32], was highly fruitful and stimulated the prediction of stable or at least metastable fundamental light bullets in various static periodic systems, such as optical lattices [33, 34, 35, 36, 37], multidimensional discrete waveguide arrays [38, 39, 40, 41, 42], photonic crystal fibers [43, 44], as well as in multimode fibers [45, 46], graded‐index materials [47, 48, 49], and dynamical twisted or modulated waveguide arrays [50, 51]. Fundamental light bullets have been observed experimentally in photonic crystal fibers, although their parameters evolved slowly because of Raman‐induced frequency shift, eventually resulting in pulse spreading [44, 52].

The realization of self‐sustained 3D states carrying vorticity remains particularly challenging; see reviews [6, 53, 54, 55, 56]. A defining characteristic of vortex states is their helical phase profile, dictating a specific donut‐shaped intensity distribution (in a uniform medium). Due to their unique phase structure, vortex‐carrying states are of considerable interest for various practical applications ranging from information encoding to particle trapping [57, 58, 59, 60]. However, besides being vulnerable to super‐critical collapse in Kerr media, vortex‐carrying states in these media, as well as in quadratic and saturable ones, are also susceptible to azimuthal modulation instability [61, 62, 63]. Nevertheless, the utilization of optical lattices or waveguide arrays allows one to suppress even azimuthal modulation instabilities [38, 64, 65] and construct vortex bullets in the form of several strongly interacting wavepackets localized on different lattice sites with a common vortical phase imposed on them. Such light bullets, however, have very limited intervals of stability due to the nonmonotonic dependence of their energy on propagation constant in the 3D case and require high pulse energies/peak powers for their excitation. In periodic lattices, they can be stable only if their energy exceeds a certain threshold. So far, 3D vortex light bullets in periodic lattices have been observed only as transient, essentially unstable objects [66].

The above limitations can be lifted in suitably engineered photonic lattices with nontrivial geometry. In particular, it has recently been shown that aperiodic lattices containing disclination defects, which modify the global lattice geometry beyond purely local distortions, can host localized states with distinct discrete rotational symmetries and properties unattainable in periodic configurations [67, 68]. Such lattices support degenerate disclination defect modes, whose combinations allow one to construct spatial vortex states that remain localized even in the linear regime. As a consequence, *spatial* vortex solitons associated with these disclination defect modes can exist without an excitation threshold [69, 70, 71, 72]. Remarkably, the resulting broadened set of discrete rotational symmetries governs both the admissible topological charges of the solitons and their stability properties. The fundamental question of whether aperiodic lattices with a disclination can support stable and thresholdless spatiotemporal (viz. 3D) vortex‐carrying states, and how their properties are affected by the lattice symmetry, remains open.

In this work, we report on stable thresholdless vortex light bullets supported by global disclination defects introduced into 2D Su–Schrieffer–Heeger (SSH) lattices. The undeformed 2D SSH lattice is a generalization of the 1D SSH model originally introduced in solid‐state physics, which considers 1D chains with tunable intracell and intercell couplings between neighboring sites [73], where a topological phase emerges when intercell couplings become stronger than intracell ones. Its 2D generalization considered here represents square lattice with four sites per unit cell, whereas the ratio between intracell and intercell coupling strengths can be tuned via the Kekulé distortion mechanism [74, 75], enabling transitions into higher‐order topological insulator phase. Although undeformed lattices of this type are traditionally employed for the study of topological vortex‐free modes emerging in their outer corners (see, e.g., [37]), here we, in contrast, are interested in light localization in the very center of the structure caused by its global deformation via the introduction of a disclination. Vortex bullets in these aperiodic lattices with variable discrete rotational symmetry emerge from localized disclination defect modes in finite spectral gaps, whereas their available topological charges are determined by the discrete rotational symmetry of the lattice. Extended stability domains for 3D vortex bullets are obtained; they are typically located near bifurcation points from spatial defect modes. Remarkably, our system with tunable discrete rotational symmetry allows us to obtain not only stable bullets with topological charge m=±1, but also stable bullets with m=±2 and potentially even higher charges, overcoming limitations encountered in graded‐index media [49].

## 2 Theoretical Model

The paraxial propagation of spatiotemporal wavepackets in a Kerr nonlinear medium with imprinted shallow transverse refractive index modulation is described by the dimensionless nonlinear (3 + 1)D Schrödinger equation

(1) $$i\frac{\partial \psi }{\partial z}=-\frac{1}{2}\nabla_{⊥}^{2}\psi -\frac{1}{2}\frac{\partial^{2}\psi }{\partial t^{2}}-R\left(x,y\right)\psi -|\psi {|}^{2}\psi ,$$

where ψ is the light field amplitude, $\nabla_{⊥}^{2}\equiv \partial_{x}^{2}+\partial_{y}^{2}$ is the transverse Laplacian, (x,y) and z are the transverse coordinates and propagation distance, which are normalized to the characteristic transverse scale $r_{0}$ and diffraction length $kr_{0}^{2}$, respectively, where $k\left(\omega \right)=2\pi n_{0}\left(\omega \right)/\lambda =n_{0}\left(\omega \right)\omega /c$ is the wavenumber, $n_{0}$ is the unperturbed refractive index, and λ is the wavelength. Normalized time $t=\left(T-kr_{0}^{2}z/v_{g}\right)/T_{s}$ is defined in the comoving frame with the group velocity $v_{g}$ [76], where characteristic time scale is $T_{s}={\left[-\beta_{2}k\left(\omega \right)r_{0}^{2}\right]}^{1/2}$, and we assume anomalous group velocity dispersion $\beta_{2}\equiv \partial^{2}k/\partial \omega^{2}<0$ at working frequency. The function

$$R\left(x,y\right)=p\sum_{m,n}e^{-\left[{\left(x-x_{m,n}\right)}^{2}+{\left(y-y_{m,n}\right)}^{2}\right]/\sigma^{2}}$$

describes the refractive index distribution in the lattice composed of identical Gaussian waveguides of depth $p=k^{2}r_{0}^{2}\delta n/n_{0}$, with δn being the refractive index contrast, width σ, and coordinates of centers $\left(x_{m,n},y_{m,n}\right)$. Such optical potentials can be fabricated using fs‐laser writing in fused silica slabs [69, 77, 78, 79, 80, 81, 82, 83, 84] and by means of other techniques, see experiments in [85, 86]. In this case, using characteristic transverse scale $r_{0}=20\;\mu m$, one obtains that dimensionless lattice depth p=10 corresponds to $\delta n\approx 10^{-3}$ at λ=1550nm [52], z=1 corresponds to about 2.35mm of propagation. The group velocity dispersion of fused silica at this wavelength is $\beta_{2}\approx -28\;{fs}^{2}/mm$ and thus $T_{s}=8.1\;fs$. According to our estimates, the dimensionless amplitudes |ψ|∼1 of light bullets reported below correspond to peak powers ∼1 MW that is well below a material damage threshold for obtained pulse durations.

Alternative implementations of such lattice potentials are also possible with other photonic platforms. For example, the experimental observation of fundamental light bullets was reported in a lattice of evanescently coupled single‐mode fiber waveguides fabricated using the stack‐and‐draw technique [52]. This fabrication technique can be adapted for fabrication of structures containing disclinations and other defects. Another promising platform is provided by optofluidic waveguides and selectively liquid‐filled photonic crystal fibers, where the nonlinear response of the infiltrating material can significantly exceed that of silica. This enhanced nonlinearity enables nonlinear localization at reduced power levels and over shorter interaction lengths, thereby facilitating the observation of spatiotemporal localized states under more accessible experimental conditions [87, 88, 89, 90]. Importantly, such materials may also be used to control the effective dispersion of the medium. Stationary 3D solutions of Equation (1), including vortex‐carrying ones, can be found in the form $\psi =u\left(x,y,t\right)e^{ibz}$, which leads to the equation

(2) $$bu=\frac{1}{2}\left(\nabla_{⊥}^{2}+\frac{\partial^{2}}{\partial t^{2}}\right)u+R\left(x,y\right)u+|u{|}^{2}u,$$

for profile u of the nonlinear self‐sustained state, where b is the propagation constant of this state. At low field amplitudes, when nonlinearity is negligible, the same equation with omitted dispersion $\left(\partial_{t}^{2}u\right)$ and nonlinear $\left(|u{|}^{2}u\right)$ terms can be used for the calculation of time‐independent spatial (2D) eigenmodes of the optical potential R(x,y).

## 3 Lattices and Modes

We consider a disclination lattice engineered from the 2D SSH lattice with $C_{4}$ discrete rotational symmetry depicted in Figure 1a. In this lattice, the intracell spacing $d_{1}$ and intercell spacing $d_{2}$ between neighboring sites are made tunable, so that $d_{1}+d_{2}=2a$, where 2a is the width of the unit cell. As previously stated, we adopt the well‐known Kekulé distortion [75, 91] (shift of waveguides in each unit cell) to control the spectrum of eigenmodes of the lattice (also known as the Wu‐Hu approach [74]). This distortion is quantified by the parameter $\delta =d_{1}/a$. Next, we insert a single Frank sector (highlighted with a blue background) into the 2D SSH lattice, and shift waveguides to accommodate the added sector. This creates the lattice with $C_{5}$ discrete rotational symmetry and global disclination defect. Notice that we do not remove the central cell of the lattice. Two representative disclination lattices corresponding to δ=0.8 and δ=1.45 are shown in Figure 1a.

**FIGURE 1:**
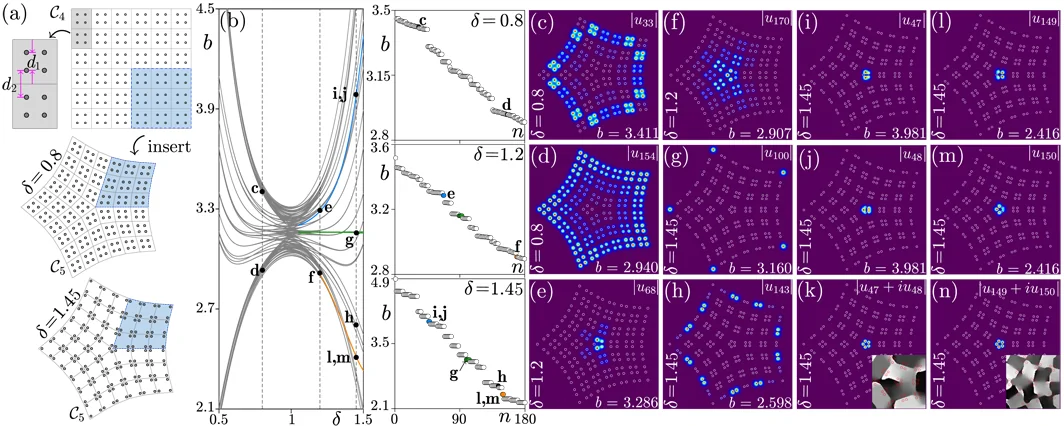
(a) $C_{4}$‐symmetric two‐dimensional SSH lattice and $C_{5}$‐symmetric disclination lattices with different distortion parameters δ constructed on the basis of $C_{4}$ structure. (b) Linear spectrum of the $C_{5}$‐symmetric disclination lattice as a function of δ (left) and eigenvalues $b_{n}$ versus mode index n (right) corresponding to three vertical dashed lines in the left panel. Dots with letters correspond to representative eigenmodes $u_{n}$, whose $\left|u_{n}\right|$ distributions (with superimposed on them array contours) are depicted in panels (c‐n). Degenerate disclination states $u_{47}$ (i) and $u_{48}$ (j) that can be used to construct vortex state $u_{47}+iu_{48}$, whose modulus and phase are shown in (k). Degenerate disclination states $u_{149}$ (l) and $u_{150}$ (m) that can be used to construct vortex state $u_{149}+iu_{150}$ shown in (n). Modes in panels (c–n) are shown within the window −21≤x,y≤21. Other parameters are a=2.5, σ=0.5, and p=10.

The propagation constants b of all linear spatial eigenmodes supported by such lattices versus the distortion parameter δ are shown in Figure 1b, left panel. It should be stressed that the introduction of the disclination defect modifies the nearest‐neighbor couplings in the entire continuous lattice in comparison with the original 2D SSH lattice, where intracell and intercell couplings are the same in the entire structure. As a result, bands corresponding to bulk and edge states experience certain splitting, as illustrated by the gray lines in Figure 1b. Still, in the spectrum, one clearly resolves groups of eigenvalues corresponding to disclination defect states residing in the center of the structure (blue and orange lines) and topological states in the outer corners (green lines). To illustrate the details of the spectrum, we plot propagation constants $b_{n}$ of all eigenmodes versus mode index n in the right‐hand panels of Figure 1b. The three panels correspond to representative distortion parameter values δ=0.8, δ=1.2 and δ=1.45 (vertical dotted lines in the left panel). For δ=0.8, all states are delocalized, see examples in Figure 1c,d. At δ=1.2, we select two disclination states $u_{68}$ (letter e) and $u_{170}$ (letter f), which correspond to different (nondegenerate) propagation constants b, and show their profiles in Figure 1e,f. These disclination defect modes are weakly localized, and even though they reside in the center of the lattice, they extend across many cells. When the distortion parameter is increased to δ=1.45, the localization of disclination modes dramatically increases, see degenerate states $u_{47}$ (letter i) and $u_{48}$ (letter j) in Figure 1i,j, respectively. Such states coexist with strongly localized corner states ($u_{100}$, letter g) and edge states ($u_{143}$, letter h), shown in Figure 1g,h, respectively. Linear combination $u_{47}\pm iu_{48}$ produces a disclination vortex state with topological charge m=±1 [see modulus and phase distribution in Figure 1k], whereas linear combination $u_{149}\pm iu_{150}$ of the second pair of degenerate disclination defect states, $u_{149}$ and $u_{150}$ [see Figure 1l,m], produces disclination vortex with m=±2, see Figure 1n.

Such modes (independent of time t) can give rise to 3D disclination vortex light bullets localized in both space and time. The latter can bifurcate (when nonlinearity and dispersion come into play) from linear localized states in spectral gaps of the system.

## 4 Vortex Light Bullets and Stability Analysis

To numerically obtain 3D disclination vortex light bullets, we solve Equation (2) iteratively by means of the modified squared‐operator method [92], with the initial ansatz in the form of a disclination vortex state with a Gaussian envelope in time t. The peak amplitude A=max|ψ| and total energy $U=∭|\psi {|}^{2}\;dxdydt$ of the obtained disclination vortex bullets are presented in Figure 2a,b, respectively, as functions of propagation constant b. The vertical dotted blue and orange lines indicate the eigenvalues of linear 2D spatial vortex modes from which bifurcation occurs. Remarkably, vortex light bullets are thresholdless, their peak amplitude and energy vanish when $b\to b_{n}$, where $b_{n}$ is the eigenvalue of the corresponding degenerate spatial modes giving rise to the vortex. We choose four vortex bullets (two stable and two unstable ones) with m=+1 and m=−2, as marked by colored dots with letters in Figure 2a,b, and show corresponding isosurfaces of |ψ| in Figure 2c–f, respectively. Spatial modulus and phase (insets) distributions in the t=0 cross‐section are shown beneath each isosurface plot, whereas bottom panels show temporal bullet profiles at x,y point corresponding to the maximum of the field amplitude. One finds that farther from the bifurcation point, the temporal profile becomes narrower, whereas spatial localization moderately increases with b because it is mainly dictated by the degree of confinement of the spatial vortex mode from which bifurcation occurs. At $b\to b_{n}$, as peak amplitude decreases, the temporal distribution tends to a uniform one. Remarkably, bullets are found in finite spectral gaps of the system. As their propagation constant b increases, it may cross eigenvalues of multiple linear modes (gray regions), but coupling with such modes does not occur, at least for the range of b values shown in Figure 2. $C_{5}$ discrete rotational symmetry of the present lattice allows coexistence of bullets with topological charges m=±1,±2, but not with higher ones, as the latter are prohibited by the symmetry of the lattice.

**FIGURE 2:**
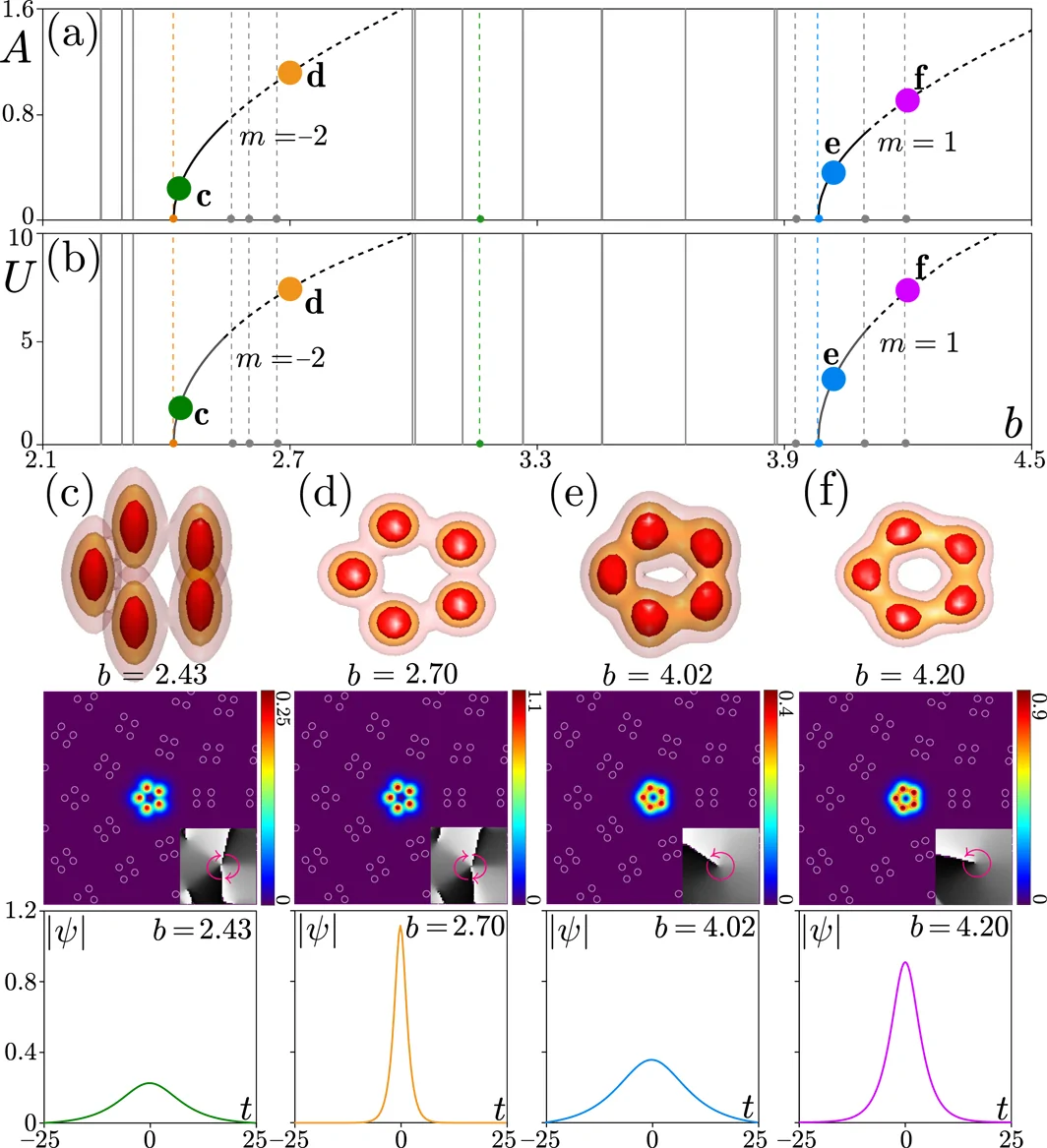
Peak amplitude A (a) and total energy U (b) of the disclination vortex bullets as functions of propagation constant b at δ=1.45. Solid (dashed) lines correspond to stable (unstable) bullets. Gray regions indicate eigenvalues corresponding to groups of extended spatial eigenmodes. Vertical orange and blue lines correspond to eigenvalues of disclination states. (c‐f) Isosurface plots (top row) of |ψ| corresponding to the dots marked with letters (c‐f) in panels (a), (b). Isosurface plots correspond to the |ψ|=0.4A, 0.6A, and 0.8A, levels. Panels with a purple background show the field modulus and phase distributions in t=0 cross‐section within the window −10≤x,y≤10. Bottom row shows temporal bullet profiles in the x,y point corresponding to |ψ|=A.

To study the stability of the vortex light bullets, we used direct evolution, when 5% (in amplitude) broadband noise is superimposed on the input bullet, and the latter is allowed to propagate up to z∼5000 in accordance with Equation (1). Such distances are typically sufficient to detect any deformations that may be caused by the development of potential (even very weak) instabilities, or prove that the bullet is stable and maintains its internal structure (including vortical phase). In Figure 2a,b, stable and unstable bullet branches are shown with solid and dashed lines, respectively. It is important that stability can be achieved even for low‐energy bullets, that is, exceeding threshold energy is not required for stability (as it happens with light bullets supported by static periodic lattices). For the stable vortex bullet with m=−2 corresponding to point **c** in Figure 2, the peak amplitude of the perturbed field only slightly oscillates upon propagation, as shown by the green line in Figure 3a. The isosurface plots of this perturbed bullet and phase distributions at t=0 shown in Figure 3b for selected propagation distances [marked by black dots in corresponding A(t) dependence] show no visible distortions (except for obvious rotation of phase due to accumulated phase shift upon propagation), further confirming stability of this state. The evolution of the unstable perturbed vortex bullet corresponding to point **d** in Figure 2 is presented in Figure 3c. As the propagation distance increases, the five interacting “pearls” constituting the bullet start to oscillate strongly, and the phase also undergoes considerable variations, as shown in Figure 3d. Vortex light bullets with topological charge m=1 exhibit qualitatively similar stability properties. They are stable in the region adjacent to the bifurcation point from spatial states [see stable dynamics illustrated in Figure 3e,f corresponding to point **e** in Figure 2], but become unstable sufficiently far from it [see dynamics in Figure 3g,h corresponding to point **f** in Figure 2]. Remarkably, both m=1 and m=2 nonlinear states can be stable in the same $C_{5}$ system, since the discrete rotational symmetry of the lattice usually imposes severe restrictions on the topological charges of stable modes.

**FIGURE 3:**
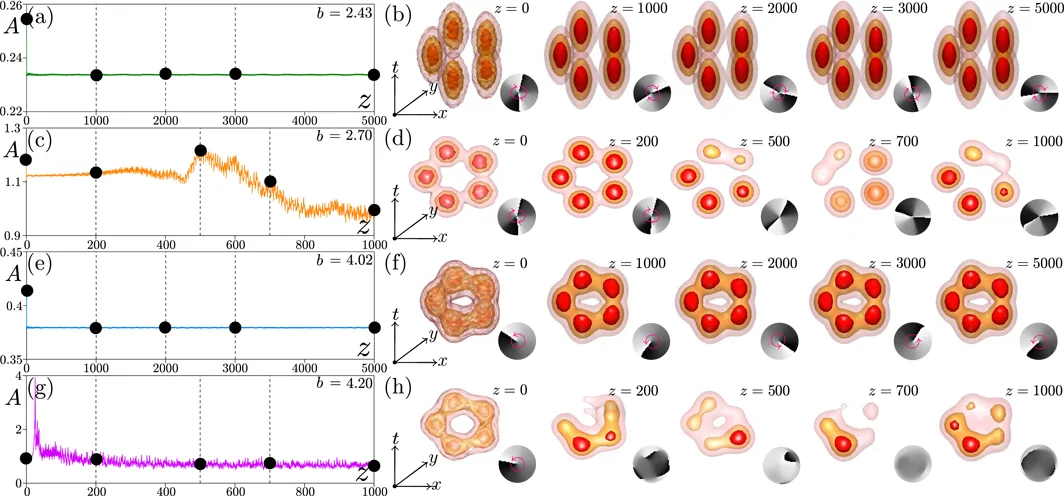
Propagation dynamics of the disclination vortex bullets. (a) Peak amplitude A of the bullet versus propagation distance z for the state corresponding to point **c** in Figure 2, and (b) representative isosurfaces (phase at t=0 is shown in the insets) at different propagation distances corresponding to black dots in (a). Respectively, panels (c), (d) show evolution of bullet **d**, panels (e), (f) show evolution of bullet **e**, whereas panels (g), (h) show evolution of bullet **f** from Figure 2.

To demonstrate the generality of our findings, we show that vortex light bullets are not specific to the $C_{5}$‐symmetric configuration, but arise in disclination lattices with different discrete rotational symmetries, which determine the allowed values of topological charge m. In particular, $C_{3}$ and $C_{6}$‐symmetric lattices that can be constructed from the original $C_{4}$ SSH lattice support various spatial disclination vortex states, from which vortex light bullets can bifurcate. As representative examples, we consider a $C_{3}$‐symmetric lattice with δ=1.6 [Figure 4a], supporting only vortex bullets with m=±1, an example of which is presented in Figure 4b, and a $C_{6}$‐symmetric lattice with δ=1.35 [Figure 4c], which hosts disclination vortex states with m=±1 and m=±2, whose examples are presented in Figure 4d and 4e, respectively.

**FIGURE 4:**
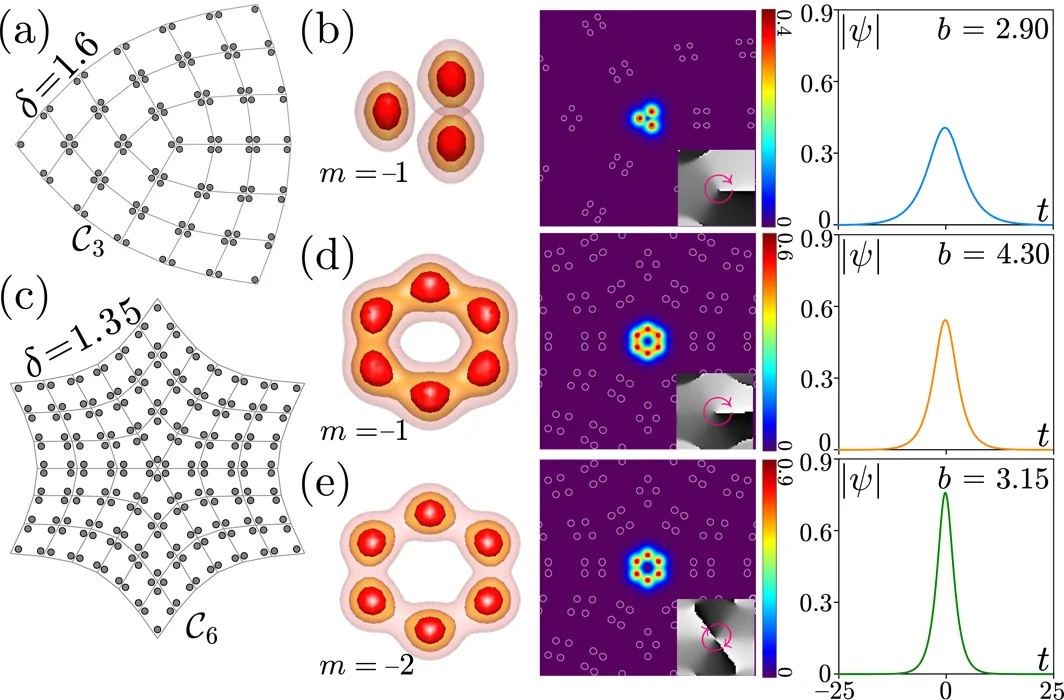
$C_{3}$‐symmetric (a) and $C_{6}$‐symmetric (c) disclination lattices for different values of the distortion parameter δ indicated near the lattice profile. Isosurface plots of exemplary disclination vortex bullets in $C_{3}$ (b) and $C_{6}$ (d), (e) lattices. Panels with a purple background show field modulus and phase (insets) distributions in the cross‐section t=0, whereas temporal profiles at coordinates x,y, corresponding to the maximum of the bullet amplitude, are shown in the right column. The values of propagation constants are indicated on temporal profiles.

More generally, the accessible topological charges are fundamentally limited by the discrete rotational symmetry of the system [93, 94]. Realizing higher‐charge vortex states would therefore require increasing the symmetry order by introducing additional Frank sectors into the disclination geometry. Such strongly modified lattices are structurally more complex, and resulting vortex solitons are typically less robust, as higher‐charge states are increasingly susceptible to azimuthal instabilities. Finally, we note that, in addition to vortex‐carrying states, all the lattices mentioned above support trivial‐phase bullets with m=0 that bifurcate from vortex‐free spatial disclination states (that are always nondegenerate). Such states can also be dynamically stable, but since we are interested primarily in excited vortex bullets, we do not show them here.

## 5 Conclusions

Summarizing, we have reported on stable 3D vortex light bullets that bifurcate from the 2D linear vortex‐carrying defect states in lattices with a global disclination defect introduced by removing or inserting Frank sectors into the SSH structure. The available topological charges of these vortex bullets are determined by the discrete rotational symmetry of the underlying disclination lattice. The remarkable result is that vortex light bullets, guided by the disclination defects, can be stable over extended intervals of propagation constants in finite spectral gaps. Moreover, light bullets with topological charges m=1 and 2 can be simultaneously stable, at least in the $C_{5}$ lattice. The stability of the bullets reported here does not require exceeding the threshold energy. This hints at enhanced robustness of such bullets that should survive even in the presence of higher‐order temporal effects or weak losses. Our results suggest that aperiodic optical lattices with global defects can offer rich prospects for engineering stable multidimensional states and pave the way for advanced optical manipulation of them.

An important question concerns the experimental realization of the predicted vortex light bullets. One of the most accessible platforms is represented by femtosecond laser‐written waveguide arrays in fused silica. This technology enables the fabrication of 2D waveguide lattices with high precision in the positioning of individual waveguides, allowing accurate control of both the lattice geometry and the coupling landscape. Such structures have already been employed for the experimental observation of spatial nonlinear topological states, including 2D vortex solitons localized at disclination defects [72]. In addition, waveguide arrays inscribed in fused silica offer a favorable combination of self‐focusing Kerr nonlinearity, anomalous group‐velocity dispersion within appropriate wavelength range, and the possibility to engineer diffraction, making them particularly well suited for the formation and observation of spatiotemporal localized states.

Alternative implementations may also be envisaged in other photonic platforms. In particular, photonic crystal fibers provide a flexible route toward realizing structured optical lattices and defect‐engineered geometries. One promising fabrication approach is the stack‐and‐draw technique, which enables the creation of fibers with characteristic feature sizes reaching the submicron regime, including hole diameters on the order of 100 nm [95, 96]. A variety of methods have been developed for selectively infiltrating such structures with liquids, liquid crystals, and solid materials, thereby providing extensive control over their optical properties [97]. Of particular interest for the present work is the possibility of filling selected channels with highly nonlinear materials, whose nonlinear coefficients can substantially exceed those of fused silica [89, 90]. This allows to enhance nonlinear interactions and control effective dispersion of the material that may facilitate the formation of spatiotemporal localized states. These possibilities suggest that light bullets reported here are not limited to laser‐written structures and may be explored across a broad range of micro‐ and potentially nano‐structured photonic architectures.

## Author Contributions

**Zhuo Zhang:** investigation, formal analysis, data curation. **Sergey K. Ivanov:** supervision, writing – review and editing, writing – original draft, formal analysis, funding acquisition, investigation. **Yongdong Li:** supervision. **Yaroslav V. Kartashov:** conceptualization, supervision, formal analysis, writing – review and editing, writing – original draft, funding acquisition, investigation. **Yiqi Zhang:** conceptualization, investigation, funding acquisition, writing – original draft, formal analysis, supervision, writing – review and editing.

## Funding

This work was supported by the Natural Science Basic Research Program of Shaanxi Province (Grants 2024JCJCQN‐06, 2025JC‐QYCX‐006), the National Natural Science Foundation of China (Grant 12474337), the Key Research and Development Program of Shaanxi Province (Grant 2025CY‐YBXM‐037), the Postdoctoral Research Project of Shaanxi Province (Grant 2023BSHYDZZ14), the Sichuan Science and Technology Program (Grant 2025ZNSFSC1458). Y.V.K. acknowledges funding by the Russian Science Foundation (Grant 24‐12‐00167) and partially by the Project FFUU‐2024‐0003 of the Institute of Spectroscopy of RAS. S.K.I. acknowledges funding from the European Union Through the Program Fondo Social Europeo Plus 2021‐2027(FSE+) of the Valencian Community (Generalitat Valenciana CIAPOS/2023/329).

## Data Availability

The data that support the findings of this study are available from the corresponding author upon reasonable request.
